# L-Arginine Supplementation for Nulliparous Sows during the Last Third of Gestation

**DOI:** 10.3390/ani11123476

**Published:** 2021-12-06

**Authors:** Gustavo de Amorim Rodrigues, Dante Teixeira Valente Júnior, Marcos Henrique Soares, Caroline Brito da Silva, Fernanda Abranches Fialho, Lívia Maria dos Reis Barbosa, Mariana Machado Neves, Gabriel Cipriano Rocha, Marcio de Souza Duarte, Alysson Saraiva

**Affiliations:** 1 Department of Animal Sciences, Universidade Federal de Viçosa, Viçosa 36570-900, MG, Brazil; gustavo.a.rodrigues@ufv.br (G.d.A.R.); dante.junior@ufv.br (D.T.V.J.); marcos.henrique@ufv.br (M.H.S.); caroline.brito@ufv.br (C.B.d.S.); fernanda.abranches@ufv.br (F.A.F.); liviamreisbarbosa@gmail.com (L.M.d.R.B.); machadoneves@gmail.com (M.M.N.); gcrocha@ufv.br (G.C.R.); 2 Muscle Biology and Nutrigenomics Laboratory, Department of Animal Sciences, Universidade Federal de Viçosa, Viçosa 36570-900, MG, Brazil; 3 Structural Biology Laboratory, Department of Animal Sciences, Universidade Federal de Viçosa, Viçosa 36570-900, MG, Brazil; 4 Department of Animal Biosciences, University of Guelph, Guelph, ON N1G-2W1, Canada; mduarte@uoguelph.ca

**Keywords:** functional amino acids, fetal development, myogenesis, skeletal muscle

## Abstract

**Simple Summary:**

Nutrition of gestating sows plays an important role in the uteroplacental efficiency, muscle development, birth weight and viability of the piglets. This is especially important for nulliparous sows, since the reproductive performance of nulliparous sows is lower compared to multiparous ones. Previous studies have shown that arginine enhances angiogenesis and placental growth, being beneficial to fetus growth. Because during its formation process skeletal muscle tissue compete for nutrients with other organs also in formation during the intrauterine development, the increase of nutrient flow by enhancing angiogenesis may benefit the skeletal muscle formation during the fetal stage. In this sense, arginine supplementation for sows seems to be a promising nutritional strategy to maximize myogenesis and muscle development of piglets. In the present study, piglets born from sows fed diet with 1.0% L-arginine (ARG) had greater mRNA expression of the gene encoding myoblast determination protein (MYOD) and myogenin (MYOG) compared to the piglets born from sows at the control group. The mRNA expression of IGF-2 gene tended to be greater in piglets born from ARG sows compared to those born from control sows. Despite differences in gene expression, no differences in the histomorphometric variables of skeletal muscle were observed between piglets from arginine-supplemented and control sows. In conclusion, supplementation of 1.0% L-arginine for nulliparous sows from 85 to 114 days of gestation increased mRNA expression of the myogenic regulatory factors MYOD and MYOG and IGF-2 in skeletal muscle of piglets.

**Abstract:**

We evaluated the effects of L-arginine supplementation during the last third of gestation on molecular mechanisms related to skeletal muscle development of piglets and litter traits at birth. Twenty-three nulliparous sows averaging 205.37 ± 11.50 kg of body weight were randomly assigned to the following experimental treatments: control (CON), where pregnant sows were fed diets to meet their nutritional requirements; arginine (ARG), where sows where fed CON + 1.0% L-arginine. Skeletal muscle from piglets born from sows from ARG group had greater mRNA expression of *MYOD* (*p* = 0.043) and *MYOG* (*p* ≤ 0.01), and tended to present greater mRNA expression (*p* = 0.06) of *IGF-2* gene compared to those born from CON sows. However, there were no differences (*p* > 0.05) in the histomorphometric variables of fetuses’ skeletal muscle. The total weight of born piglets, total weight of born alive piglets, piglet weight at birth, coefficient of variation of birth weight, and the incidence of intrauterine growth restriction (IUGR) piglets did not differ between groups. No stillborn piglets (*p* < 0.01) were verified in the ARG sows compared to CON group. The blood levels of estradiol (*p* = 0.035) and urea (*p* = 0.03) were higher in ARG sows compared to those from the CON group. In summary, our data show that arginine supplementation of nulliparous sows at late gestation enhance mRNA expression of key myogenic regulatory factors, which likely contribute to improve animal growth rates in later stages of development.

## 1. Introduction

In the last few decades, the genetic breeding industry has focused on increasing the production capacity of the sows. However, the increase in the number of born piglets has negatively impacted uteroplacental efficiency, impairing the distribution of nutrients and oxygen from the mother to the fetuses, influencing prenatal muscle development and resulting in greater numbers of low viability piglets [1,2,3]. Because the birth weight of the progeny of nulliparous sows is lower compared to multiparous ones, parity order must also be considered an important factor that affects the intrauterine development of fetuses. Therefore, studies to evaluate nutritional strategies to maximize prenatal muscle development in fetuses of nulliparous sows are needed.

Arginine is a precursor of biologically active molecules, such as nitric oxide and polyamines, key factors in angiogenesis and placental growth [4,5]. Therefore, dietary supplementation with arginine has been evaluated as a potential alternative to improve fetal muscle development in pigs [6,7,8]. Previous studies have demonstrated that arginine supplementation alters the expression of myogenic regulatory factors (MRFs) and insulin-like growth factors (IGFs) in skeletal muscle by changing protein synthesis, cell proliferation, differentiation, and growth [9,10,11,12,13].

Supplementation with L-arginine, from 14 to 28 days of gestation, has positively affected fetal myogenesis promoting greater hyperplasia of primary muscle myofibers [6], greater formation of secondary myofibers [8] and alterations in the expression of MRFs and *IGF-2* in the skeletal muscle of piglets [11] at 75 days of gestation.

However, studies with arginine supplementation in late gestational period are scarce. Because the at final third of gestation (from 85 days), the skeletal muscle is more susceptible to factors related to the uterine environment, in addition to of being the period when fetal growth increases exponentially, nutritional strategies at this stage of gestation may enhance the rate of protein deposition in the fetuses [14,15].

Thus, we hypothesized that supplementation with 1.0% L-arginine, from 85 days of gestation until parturition, may enhance fetal myogenesis and improve piglet muscle development and litter traits at birth. Therefore, this study was carried out to evaluate the effects of L-arginine supplementation during the final third of gestation on mRNA expression of key genes regulating myogenesis, muscle development of piglets, and litter traits at birth.

## 2. Materials and Methods

### 2.1. Animals, Experimental Design and Diets

Twenty-three commercial hybrid nulliparous sows with initial weight of 205.37 kg ± 11.50 kg were used, housed in individual stalls (2.29 m × 2.17 m) from 85 to 110 days of gestation and in farrowing cages (2.40 m × 0.60 m) from 110 days of gestation to farrow.

Sows were randomly assigned in a completely randomized experimental design, with 12 and 11 replicates for control (CON) and L-arginine (ARG) diets, respectively. CON diet was formulated according to Rostagno et al. [16] and ARG diet was obtained by adding 1.0% L-arginine to the CON in place of inert clay filler. The level of 1.0% L-arginine was chosen based on previous studies and on its ability to increase plasma concentration of arginine in sows [17]. In order to avoid competition for basic amino acid transporters and toxicity, it was sought not to exceed the 2.0% arginine in both diets and not to exceed the SID arginine to SID lysine ratio of 3.0 [5].

Experimental diets (Table 1) were provided from 85 days of pregnancy until parturition in equal amounts at 9:00 A.M. and 4:00 P.M., totaling 2.5 kg/day. The sows had free access to water throughout the experimental period and consumed all the feed offered. The temperature and humidity inside the facility were daily recorded using a data logger device (Akso, São Leopoldo, Brazil).

### 2.2. Blood Sampling and Analysis

At 110 days of gestation, sows were weighed and blood samples were collected in two 10 mL tubes (Becton Dickinson Vacutainer Systems), one with silicone and the other with EDTA (ethylenediamine tetraacetic acid) to obtain serum and plasma, respectively. One hour after collection, blood samples were centrifuged (Eppendorf^®^ 5702R, Eppendorf AG, Hamburg, Germany) at 3000× *g* for 10 min at 4 °C, allowing the removal of serum and plasma, which were transferred to 1.5 mL microtubes and stored at −20 °C until further analysis.

Serum samples were used to determine the concentrations of urea (Ureal Cobas c 311; Roche Diagnostics GmbH, Basel, Switzerland) and insulin (Kit Architect Insulina; Abbott Laboratórios do Brasil Ltd.a., São Paulo, Brazil). Plasma samples were used to determine estradiol concentration using microparticle chemiluminescence immunoassay (Architect estradiol assay, Abbott, IL, USA).

### 2.3. Litter Traits

The number of total born, born alive, stillborn and mummified piglets was recorded. After birth, sows and piglets were weighed to calculate total weight of born piglets, total weight of born alive piglets, mean birth weight, standard deviation of mean birth weight, coefficient of variation of mean weight at birth and distribution of piglets in weight classes: <1.00 kg; 1.00 to 1.20 kg; 1.20 to 1.45 kg; 1.45 to 1.70 kg; >1.70 kg. Piglets weighing less than 1.0 kg at birth were considered as intrauterine growth restricted (IUGR) [18].

### 2.4. Piglet Skeletal Muscle Tissue Sampling

Twelve hours after farrowing, one piglet per litter with body weight closest to the average weight of its respective litter was electrically stunned followed by exsanguination for sampling *Longissimus dorsi* and *Semitendinosus* muscles for histological and molecular analysis.

The samples for histological analysis (2 cm × 3 cm sections) were placed in 50 mL vials with 4% paraformaldehyde in a 0.1 M phosphate buffer solution and incubated overnight at room temperature for 24 h. After this period, the samples were preserved in 70% ethanol until processing and histological cross sections.

A subset of samples was collected at slaughter, immediately after exsanguination, and snap-frozen in liquid nitrogen. Frozen samples were then stored at −80 °C until the RNA extraction process.

### 2.5. Histomorphometric Analysis of Piglet Skeletal Muscle Tissue

*Longissimus dorsi* and *Semitendinosus samples* previously fixed and stored in 70% ethanol were embedded in 2-hydroxyethyl methacrylate (Historesina^®^-Leica Biosystems, Buffalo Grove, IL, USA), according to the manufacturer’s recommendations. The embedded tissue was cross-sectioned at 3 µm and slides were stained with hematoxylin–eosin (HE) solution and mounted with Entellan^®^ (Merck, Frankfurt, Germany). Images of the slides was captured using a light microscope (BX53; Olympus, Tokyo, Japan), at 20× magnification, with a CMOS 1.3 MP BioCAM camera (Takachiho, Japan). A total of eight images of each tissue per piglet were recorded to determine the volumetric proportion of muscle tissue components and the number of muscle fibers, which were measured using the Image J^®^ program (version 1.50i; National Institutes of Health, Bethesda, MD, USA). The total number of muscle fibers in the tissues was evaluated by counting each histological image (area: 0.131 mm^2^). The volumetric proportion of muscle tissue components was obtained by projecting 266 intersections (points) onto each histological image. Coincident points were registered and defined as nucleus, sarcoplasm and connective tissue. The percentage of points for muscle tissue was calculated using the formula: volumetric proportion (%) = (number of points in each structure/total points in muscle tissue) × 100.

### 2.6. RNA Extraction, cDNA Synthesis and RT-qPCR Assessment

Total RNA extraction was performed from 70 mg of *Longissimus dorsi* muscle samples (previously powdered in liquid nitrogen) using TRIzol^®^ (InvitrogenTM, Carlsbad, CA, USA), according to the manufacturer’s instructions. The final precipitate was rehydrated with 30 µL of UltraPure^®^ DNase/RNase-Free water. RNA concentration was estimated by spectrophotometry using the NanoDrop spectrophotometer (ThermoFisher, Waltham, MA, USA), observing the A260/A280 ratios between 1.8 and 2.0 as a purity control. The integrity of extracted RNA was verified using 1% agarose gel. Next, the samples were reverse transcribed into complementary DNA (cDNA) using the GoScript Reverse Transcription (RT) Kit (Promega, Madison, WI, USA), following the manufacturer’s recommendation.

Primers for amplification of endogenous and target gene fragments were designed using the PrimerQuest program provided by Integrated DNA Technologies, Inc. (Coralville, IA, USA) from nucleotide sequences obtained from the GeneBank database (http://www.ncbi.nlm.nih.gov (accessed on 14 September 2019)) (Table 2).

The genes, *ACTB*, *HPRT1* and *GAPDH* were used in normalization analysis in order to minimize possible variations in the amount of initial mRNA and reverse transcription efficiency. The choice of the endogenous gene was based on the amplification efficiency of the candidate genes from the calculation of the efficiency for each primer pair (at concentrations of 100, 200 and 400 mM) using the formula E = 10^(−1/line slope)^ − 1, where E is the reaction efficiency [19] which, for the tested endogens, ranged from 0.94 to 1.22. In addition, the profile of the amplification and dissociation curves, and the amplification stability of these genes between groups were also used as a parameter. The endogenous genes showed no significant difference between groups, so the *GAPDH* gene was chosen.

The RT-qPCR analyses were performed in duplicate using the QuantStudio 3 thermocycler (Applied Biosystems, Foster City, CA, USA) by the Relative Quantitation method, using as detection the SYBR^®^ Green system (Applied Biosystems, Foster City, CA, USA) and the GoTaq^®^ qPCR Master Mix kit (Promega corporation, Madison, WI, USA). The PCR reactions were submitted to the cycle protocol according to the program: 95 °C for 3 min, 40 cycles of 95 °C for 15 s and 60 °C for 1 min. The threshold cycle (Ct) values obtained were later normalized using the delta-ct method (ΔCt) based on the Ct values obtained for the endogenous control gene (*GAPDH*). The calculation of relative gene expression levels was developed according to the 2^−ΔΔCt^ method, described by Livak and Schmittgen [20].

### 2.7. Statistical Analysis

Statistical analysis was performed using public domain software R (version 3.4.3, 2017, R Foundation for Statistical Computing, Vienna, Austria, https://www.r-project.org/, accessed on 11 November 2021). Each sow was considered as an experimental unit for analysis. Data on blood, histomorphometry, gene expression and phenotypic parameters related to the sow (weight at 85 days, weight at 110 days and weight at birth) and litter (total weight, mean weight, standard deviation of the mean, coefficient of variation of the mean birth weight) were subjected to analysis of variance (ANOVA). Data on total born, born alive, stillbirths, mummified piglets and stratification of piglets by body weight classes were analyzed using the Kruskal–Wallis test, as the residuals did not show normal probability distribution (Shapiro–Wilk: *p* ≤ 0.05). A value of *p* ≤ 0.05 was considered to indicate statistical significance and *p*-values between 0.05 and 0.10 were considered as trend.

## 3. Results

### 3.1. Thermal Environment

During the experimental period the minimum and maximum temperatures recorded inside the facilities were of 16.0 ± 1.7 °C and 23.4 ± 1.9 °C, respectively. The mean of the minimum relative humidity was 55.3 ± 6.5% and the maximum 74.8 ± 4.6%. Because the thermoneutral zone for gestating sows ranges around 15 to 22 °C with relative humidity ranges around 60 to 70% [21], sows in this were kept in thermoneutral conditions.

### 3.2. Sow and Piglet Phenotypic Data

No phenotypic differences (*p* > 0.05) were observed between CON or ARG sows (Table 3). Supplementation with L-arginine did not change the number of total born (*p* = 0.458), born alive (*p* = 0.877) and mummified piglets (*p* = 0.377). However, ARG sows had no stillborn piglets (*p* < 0.01) compared to CON. In addition, there was no effect of L-arginine supplementation on weight of total born (*p* = 0.452) and born alive piglets (*p* = 0.452), piglet weight (*p* = 0.285), number of piglets weighing less than 1.0 kg (*p* = 0.921), and on other weight classes of piglets (*p* < 0.05): 1.0 to 1, 2 kg; 1.2 to 1.45 kg, 1.45 to 1.7 kg; and greater than 1.7 kg.

### 3.3. Biochemical Analysis of Blood Parameters

At 110 days of gestation sows fed ARG had higher (*p* = 0.03) plasma concentration of estradiol and higher (*p* = 0.03) serological concentration of urea (Table 4). Serum insulin concentration did not differ (*p* = 0.446) between groups.

### 3.4. Histomorphometry of Piglet Skeletal Muscle Tissue

No differences were observed (*p* > 0.05) in *Longissimus dorsi* and Semitendinosus muscles regarding the volumetric proportion of nuclei, cytoplasm and connective tissue of piglets born from ARG sows compared to CON sows (Table 5). Furthermore, the number of piglets’ muscle cells did not differ in either muscle (*p* > 0.05) between groups in both muscles.

### 3.5. Gene Expression

Supplementation of sows from 85 to 110 days of gestation with L-arginine did not influence (*p* > 0.05) the gene expression in the muscle tissue of piglets for *mTOR*, *IGF-1*, *IGF-1R* and *IGF-2R*. However, a trend (*p* = 0.06) of increased expression of the *IGF-2* gene was observed (Table 6).

Regarding myogenic regulatory factors in the muscle tissue of piglets, greater expression of *MYOD* (*p* = 0.04) and *MYOG* (*p* < 0.01) genes was observed in piglets born from sows fed ARG. There were no differences in the expression of *MSTN* (*p* = 0.752), *MRF4* (*p* = 0.782) and *MYF5* (*p* = 0.570) genes between CON and ARG groups.

There were no differences between CON and ARG sows for the expression of genes related to degradation proteins: *CASP3* (*p* = 0.866), *Atrogin-1* (*p* = 0.921), and *MURF-1* (*p* = 0.613).

## 4. Discussion

During myogenesis, from the formation of secondary fibers, factors, such as sow age, parity order, genotype, placental efficiency, vascularization at the maternal-fetal interface and metabolic and hormonal state of the embryo can affect fetal muscle development [22,23,24]. Skeletal muscle, in turn, has a lower priority in nutrient partition during fetal development than the neural system, development of internal organs and bones, and is therefore influenced by nutrient fluctuations [15]. In this sense, as suggested by Nissen et al. [25], in young sows the nutrient requirements at the end of pregnancy for fetal myogenesis may not be met, resulting in impaired muscle tissue development, compared to 3rd and 4th parity sows. 

Prenatal muscle development is regulated by a range of factors, such as myogenesis regulatory factors, MRFs (*MYF5*, *MRF4*, *MYOD* and *MYOG*), insulin-like growth factors, IGFs (*IGF-1* and *IGF-2*) and myostatin (*MSTN*) [26]. In the present study, maternal supplementation with 1.0% L-arginine resulted in increased the mRNA expression of *MYOD* and *MYOG* and components of the IGF system in the *Longissimus Dorsi* of piglets at birth. IGFs are involved in different stages of myogenesis, whether in the activation and proliferation of satellite cells or acting in the regulatory pathway of protein synthesis, stimulating cell hyperplasia and hypertrophy [27,28,29]. Furthermore, the IGF pathway can regulate MRFs family proteins, mainly *MYOD* and *MYOG*, controlling the process of proliferation, differentiation, maturation and hypertrophy of muscle fibers [30], which is consistent with the results of the present study.

During the final third of gestation in pigs, muscle development is mainly composed of the process of muscle hypertrophy, which is characterized by an increase in the size of muscle fibers, coordinated by the *mTOR* pathway mediated by *IGF-1* [31]. Aguiar et al. [32] observed a positive correlation between the increase in muscle mass in mice with the mRNA expression of *MYOD*, *MYOG* and *IGF-1*, suggesting a possible interaction of these genes in controlling muscle hypertrophy. However, in our study, although numerical differences were observed, supplementation with L-arginine did not affect the mRNA expression of *IGF-1*, *mTOR* and the sarcoplasmic proportion of the analyzed skeletal muscles which is related to the muscle fiber diameter.

However, piglets from sows fed ARG tended to have increased mRNA expression of *IGF-2*. The *IGF-2* gene is essential for myogenesis as it is mainly associated with muscle differentiation in embryos and fetuses, which is a process mediated by the *PI3K/AKT/mTOR/p70S6K* pathway [30,33]. Studies have shown that the mRNA expression of *IGF-2* increases as muscle cells become fully differentiated. This is partially due to the fact that *MYOD* promotes the induction of *IGF-2* expression after activation of the IGF signaling pathway/*AKT*. *MYOD* in the muscle differentiation phase, which is a powerful *MYOG* stimulator, which function is to promote the fusion and differentiation of myocytes and myofiber maturation, being more expressed in the middle and in the final third of myogenesis [30,33,34,35].

Thus, although we have not assessed the abundance of the proteins encoded by the candidate genes evaluated in this study, for an increased mRNA expression of *MYOD*, and *MYOG*, as well as the tendency of increase in mRNA expression of *IGF-2* in the skeletal muscle of piglets of ARG sows, suggests a prolonged period of muscle differentiation and greater maturation of myofibers. Although the hyperplasia process ceases around 95 days of gestation, genes related to myogenesis including myofiber formation may not show specific up-regulation in their expression level, but may be expressed at steady levels at birth [8].

The results of this study corroborate those of Chen et al. [12], who reported L-arginine supplementation can increase mRNA expression of *IGF-2* mRNA expression in piglet muscle tissue. Furthermore, contrary to what has been reported in other studies conducted at earlier gestational periods [8,11], L-arginine supplementation in the last third of pregnancy may influence the mRNA expression of *MYOD* and *MYOG*, regardless of piglet birth weight. Collectively, although the molecular mechanisms by which arginine regulates myofiber development are not well defined, the results of the current and previous studies demonstrates that muscle development can be influenced by the nutritional level of this amino acid.

Although molecular alterations in fetal myogenesis were observed, L-arginine supplementation did not influence the weight of total born and born-alive piglets, the average piglet weight and the coefficient of variation of piglet birth weight. Similarly, Bass et al. [36] did not observe effects on the reproductive performance of nulliparous and primiparous sows fed with 1.0% L-arginine between days 93 and 110 of gestation. In addition, there was also no reduction in the percentage of piglets characterized as IUGR (body weight <1.00 kg), and this result is in agreement with that of Quesnel et al. [37] and Hong et al. [38].

The use of nulliparous sows in the present study may partially explain the lack of arginine effects on reproductive performance traits [39]. It is noteworthy that most of the studies available in the literature in which positive effects of arginine were observed during supplementation in the final third of pregnancy have used sows with parity order greater than three [18,40,41], which may be the cause of the different responses of L-arginine of the current to previous studies. However, the lack of reproductive results may also be related to the number of sows used in this study.

Supplementation with L-arginine did not increase the number of piglets born alive. However, sows fed ARG had no stillborn piglets, a similar result to those reported by Che et al. [42] and Nuntapaitoon et al. [18]. Arginine is necessary for the placental synthesis of nitric oxide and polyamines, which stimulate not only angiogenesis and vascular growth, but also utero-placental blood flow and the transfer of nutrients and oxygen from the mother to the fetuses, providing improved uterine efficiency for fetal growth, development and survival [42,43,44,45].

Furthermore, the increase in plasma estradiol levels in sows fed ARG diet may be related to an improvement in uterine efficiency. This hormone is essential for the maintenance of pregnancy, as it is related to increased placental blood flow, by stimulating the secretion of endothelial growth factor (VEGF), which has its expression progressively increased during pregnancy in pigs [46,47]. Thus, increased plasma estradiol levels are positively correlated with the rate of fetal-placental development and fetal growth [48,49].

Diets containing high levels of crude protein and/or arginine can result in antagonism in amino acid absorption and ammonia toxicity, impairing the reproductive performance of sows [4,5,50]. In the present study, although higher concentration of urea was observed in the serum of ARG sows, this did not negatively influence the reproductive performance. Similarly, to other recent studies evaluating L-arginine supplementation for pregnant sows, without the use of isonitrogenous control, no negative effects in the reproductive performance were reported [7,18,51,52].

Maternal supplementation with L-arginine confirms the hypothesis that this amino acid, provided during the final third of pregnancy, promotes changes in fetal myogenesis, as evidenced by the increased expression of *MYOD*, *MYOG* mRNA and the increasing trend of *IGF-2* mRNA in piglet skeletal muscle.

However, the molecular mechanisms by which arginine affect the development of muscle fibers, both at cellular and molecular level, remain to be elucidated and further studies are needed to investigate to what extent the molecular alterations, observed in this study can affect postnatal growth of the piglets. On the other hand, despite the absence of stillbirths in ARG sows, the hypothesis that arginine supplementation improves muscle development, birth weight and the incidence of IUGR piglets was not confirmed. Even so, this nutritional strategy seems to be beneficial for nulliparous sows, enhancing the survival rate of piglets at birth, in addition to being able to positively influence the progeny growth potential.

## 5. Conclusions

Supplementation with 1.0% L-arginine for nulliparous sows, from 85 to 114 days of gestation, influenced the molecular mechanisms related to fetal myogenesis by increasing the mRNA expression of myogenic regulatory factors *MYOD* and *MYOG,* and *IGF-2* in the *Longissimus dorsi* of piglets. In addition, L-arginine supplementation resulted in the absence of stillborn piglets and increased plasma estradiol level in sows. However, L-arginine supplementation did not influence birth weight and the incidence of IUGR piglets, and the sarcoplasmatic proportions of the *Longissimus dorsi* and *Semitendinosus* muscle in the piglets.

## Figures and Tables

**Table 1:** Composition of experimental diets.

Ingredient, %	CON	ARG
Corn, 7.88%	67.58	67.58
Soybean meal, 45.0%	16.00	16.00
Wheat bran	12.00	12.00
Dicalcium phosphate	1.40	1.40
Inert clay filler	1.00	˗
L-arginine, 98.0%	˗	1.00
Limestone	0.750	0.750
Salt	0.340	0.340
L-lysine, 78.0%	0.215	0.215
Mineral premix ^1^	0.200	0.200
Choline chloride	0.120	0.120
Vitamin premix ^2^	0.300	0.300
L-threonine, 98.5%	0.100	0.100
Calculated nutritional composition ^3^		
SID arginine, %	0.852	1.817
Calcium, %	0.700	0.700
Metabolizable energy, kcal/kg	3145	3145
Available phosphorus, %	0.375	0.375
SID lysine, %	0.749	0.749
SID methionine + cysteine, %	0.412	0.412
Crude protein, %	14.73	17.00
Sodium, %	0.154	0.154
SID threonine, %	0.554	0.554
SID tryptophan, %	0.148	0.148

^1^ Content per kilogram of product: zinc (50 g), iron (40 g), manganese (10 g), copper (5000 mg), selenium (150 mg), iodine (141 mg), chromium (100 mg). ^2^ Content per kilogram of product: vitamin A (1,000,000 UI), vitamin D3 (1,400,000 UI), vitamin E (30,000 mg), vitamin K3 (2800 mg), vitamin B1 (1800 mg), vitamin B2 (5600 mg) vitamin B6 (1600 mg), vitamin B12 (mcg), calcium pantothenate (20 g), niacin (30 g), folic acid (1000 mg), biotin (240 mg), selenium (200 mg), butyl hydroxytoluene (5000 mg). ^3^ Values calculated according to Rostagno et al. [16].

**Table 2:** Oligonucleotides used on gene expression analysis.

Gene ^1^	GenBank Number	Sequence	Size, bp
*IGF-1*	NM_214256.1	F: GAGGCTGGAGATGTACTGTR: TCCTGAACTCCCTCTACTTG	223
*IGF-1 R*	NM_214172.1	F: ATCTGATCATCGCCCTACCR: CCCAGCCTGCTGTTATTTC	163
*IGF-2*	NM_213883.2	F: TCTCTGTACCCTTCTGTCTGR: AGAGACTAGCCTGACATGG	212
*IGF-2 R*	NM_001244473.1	F: CATCGGGAAGACCTTTGTGR: GCTCTTTCGTCTCGGTTTC	180
*MYOD*	GU249575.1	F: ACAGCGGACGACTTCTATGR: GAGTGTTCCTCGGGCTTTA	196
*MSTN*	NM_214435.2	F: GCTGTACTCCCACAAAGATGR: CACCCACAGCGATCTACTA	175
*MYOG*	NM_001012406.1	F: GGGCATGTAAGGTGTGTAAGR: CCTCAAAGGCCTCATTCAC	203
*MRF4*	DQ139775.1	F: CGCCATCAACTACATCGAGAGGTR: ATCACGAGCCCCCTGGAAT	193
*MYF5*	NM_001278775.1	F: TAGTTCCAGGCTCATCTACCR: CCTCCTTCCTCCTGTGTAA	181
*CASP3*	NM_214131.1	F: GAGGCACAGAATTGGACTGR: CAAGAAGTCTGCCTCAACTG	167
*Atrogin-1*	NM_001044588	F: TCACAGCTCACATCCCTGAGR: GACTTGCCGACTCTCTGGAC	206
*MuRF1*	NM_001184756	F: ATGGAGAACCTGGAGAAGCAR: ACGGTCCATGATCACCTCAT	158
*mTOR*	XM_003127584.6	F: GCGATAGACACCCATCTAACCR: CTTCTCTCTGGTCATAGCAACC	220
*ACTB*	XM_003124280.3	F: CTTCTAGGCGGACTGTTAGTGR: TGTCGGCGATGCCTGGGTA	123
*HPRT1*	NM_001032376.2	F: CCAGTCAACGGGCGATATAAR: GACCAAGGAAAGCAAGGTTG	165
*GAPDH*	NM_001206359.1	F: CAAAGTGGACATTGTCGCCATCAR: AGCTTCCCATTCTCAGCCTTGACT	210

^1^ *IGF-1*: insulin growth factor 1, *IGF-1R*: insulin growth factor 1 receptor, *IGF-2*: insulin growth factor 2, *IGF-2R*: insulin growth factor 2 receptor, *MYOD*: myogenic differentiation factor, *MSTN*: myostatin, *MYOG*: myogenin; *MRF4*: mitogenesis regulating factor 4, *mTOR*: mammalian target of rapamycin, *CASP3*: caspase 3, *Atrogin-1* and *MuRF1*: ubiquitin ligases E3, *ACTB*: beta-actin; *GAPDH*: Glyceraldehyde 3-phosphate dehydrogenase, *HPRT1*: hypoxanthine phosphoribosyltransferase 1. F and R indicate primers forward and reverse, respectively.

**Table 3:** Phenotypic data of sows (*n* = 23) fed CON or ARG diets and piglets.

Item	CON	ARG	SEM	*p*-Value
Sow body weight, kg				
Day 85	204.87	205.91	2.344	0.835
Day 110	228.46	227.30	2.565	0.805
Farrowing	202.32	206.27	2.604	0.299
Litter traits				
Total born ^2^	14.33	13.18	0.656	0.458
Total born alive ^2^	13.50	13.18	0.621	0.877
Stillborn ^2^	0.83	0.00	0.138	0.001
Mummified ^2^	0.50	0.18	0.149	0.377
Total birth weight, kg ^1^	20.49	18.77	0.731	0.452
Total live birth weight, kg ^1^	19.67	18.77	0.959	0.545
Piglet weight, kg ^1^	1.51	1.46	0.005	0.285
CV ^1^	18.36	16.60	2.548	0.364
Weight classes of piglets, %				
<1.0 kg ^2^	52.63	47.37	0.345	0.921
1.0 a 1.2 kg ^2^	51.28	48.72	0.354	0.774
1.2 a 1.45 kg ^2^	59.09	40.91	0.798	0.214
1.45 a 1.7 kg ^2^	50.00	50.00	0.762	0.779
>1.7 kg ^2^	55.88	44.12	0.616	0.799

^1^ Used as covariate. ^2^ Variables analyzed by the non-parametric method due to the absence of normality and/or homogeneity of variances, CV: coefficient of variation of body weight, CON: control diet; ARG: CON + 1.0% L-arginine; SEM: standard error of the mean.

**Table 4:** Blood parameters of sows (*n* = 23) at 110 days of gestation fed CON or ARG diet.

Item	CON	ARG	SEM	*p*-Value
Urea, mg/dL	21.83	25.28	2.652	0.030
Estradiol, pg/mL	517.67	638.36	28.99	0.035
Insulin, UI/mL	4.35	5.17	1.359	0.446

CON: control diet; ARG: CON + 1.0% L-arginine; SEM: standard error of the mean.

**Table 5:** Histomorphometric variables of *Longissimus dorsi* and *Semitendinosus* of newborn piglets from sows (*n* = 23) fed CON or ARG diet.

Item	CON	ARG	SEM	*p*-Value
Longissimus dorsi				
Nucleus, %	19.17	20.30	0.520	0.286
Sarcoplasm, %	33.64	32.86	0.839	0.653
Connective tissue, %	47.19	46.84	0.820	0.835
Cells number (0.131 mm^2^)	134.20	141.06	3.448	0.332
Semitendinosus				
Nucleus, %	22.08	21.85	0.473	0.823
Sarcoplasm, %	30.81	30.50	0.601	0.804
Connective tissue, %	47.11	47.65	0.516	0.528
Cells number (0.131 mm^2^)	136.95	131.65	2.880	0.370

CON: control diet; ARG: CON + 1.0% L-arginine; SEM: standard error of the mean.

**Table 6:** Fold change between diets with 1.0% L-arginine (ARG) and control (CON) fed to sows (*n* = 23) from 85 days of gestation on the relative expression of *Longissimus dorsi* genes in piglets.

Item	CON	ARG	SEM	*p*-Value
*mTOR*	1.10	1.31	0.025	0.130
*IGF-1*	1.26	1.39	0.033	0.453
*IGF-2*	1.49	1.84	0.038	0.062
*IGF-1R*	1.60	1.63	0.063	0.868
*IGF-2R*	1.35	1.46	0.028	0.508
*MYOD*	1.19	1.44	0.017	0.043
*MSTN*	2.02	1.92	0.011	0.752
*MRF4*	2.00	1.94	0.070	0.782
*MYOG*	0.78	1.08	0.009	0.003
*MYF5*	1.08	1.01	0.017	0.570
*CASP3*	1.37	1.34	0.024	0.866
*Atrogin-1*	2.57	2.52	0.366	0.921
*MURF-1*	1.88	1.75	0.067	0.613

CON: control diet; ARG: CON + 1.0% L-arginine; SEM: standard error of the mean.

## Data Availability

Data are available on request.

## References

[1] Père M.C., Etienn M. (2000). Uterine blood flow in sows: Effects of pregnancy stage and litter size. Reprod. Nutr. Dev..

[2] Bérard J., Pardo C.E., Bethaz S., Kreuzer M., Bee G. (2010). Intra-uterine crowding decreases average birth weight and affects muscle fiber hyperplasia in piglets. J. Anim. Sci..

[3] Campos P.H.R.F., Silva B.A.N., Donzele J.L., Oliveira R.F.M., Knol E.F. (2012). Effects of sow nutrition during gestation on within-litter birth weight variation: A review. Animal.

[4] Wu G., Bazer F.W., Davis T.A., Jaeger L.A., Johnson G.A., Kim S.W., Knabe D.A., Meininger C.J., Spencer T.E., Yin Y.L. (2007). Important roles for the arginine family of amino acids in swine nutrition and production. Livest. Sci..

[5] Wu G.Y., Bazer F.W., Satterfield M.C., Li X.L., Wang X.Q., Johnson G.A., Burghardt R.C., Dai Z., Wang J., Wu Z. (2013). Impacts of arginine nutrition on embryonic and fetal development in mammals. Amino Acids.

[6] Bérard J., Bee G. (2010). Effects of dietary L-arginine supplementation to gilts during early gestation on foetal survival, growth and myofiber formation. Animal.

[7] Garbossa C.A.P., Carvalho Júnior F.M., Silveira H., Faria P.B., Schinckel A.P., Abreu M.L.T., Cantarelli V.S. (2015). Effects of ractopamine and arginine dietary supplementation for sows on growth performance and carcass quality of their progenies. J. Anim. Sci..

[8] Madsen J.G., Pardo C., Kreuzer M., Beel G. (2017). Impact of dietary L-arginine supply during early gestation on myofiber development in newborn pigs exposed to intra-uterine crowding. J. Anim. Sci. Biotechnol..

[9] Yao K., Yin Y.L., Chu W., Liu Z., Deng D., Li T., Huang R., Zhang J., Tan B., Wang W. (2008). Dietary arginine supplementation increases mTOR signaling activity in skeletal muscle of neonatal pigs. J. Nutr..

[10] Kong X., Tan B., Yin Y., Gao H., Li X., Jaeger L.A., Bazer F.W., Wu G. (2012). L-arginine stimulates the mTOR signaling pathway and protein synthesis in porcine trophectoderm cells. J. Nutr. Biochem..

[11] Kalbe C., Bérard J., Porm M., Rehfeldt C., Bee G. (2013). Maternal L-arginine supplementation during early gestation affects foetal skeletal myogenesis in pigs. Livest. Sci..

[12] Chen R., Wang W., Liu S., Pan J., Li T., Yin Y. (2013). Dietary arginine supplementation altered expression of IGFs and IGF receptors in weaning piglets. J. Cell Anim. Biol..

[13] Hu C.J., Li F.N., Duan Y.H., Zhang T., Li H.W., Yin Y.L., Wu G.Y., Kong X.F. (2019). Dietary supplementation with arginine and glutamic acid alters the expression of amino acid transporters in skeletal muscle of growing pigs. Amino Acids.

[14] McPherson R.L., Ji F., Wu G., Blanton J.R., Kim S.W. (2004). Growth and compositional changes of fetal tissues in pigs. J. Anim. Sci..

[15] Du M., Wang B., Fu X., Yang Q., Zhu M.J. (2015). Fetal programming in meat production. Meat Sci..

[16] Rostagno H.S., Albino L.F.T., Donzele J.L., Gomes P.C., De Oliveira R.F.M., Lopes D.C., Ferreira A.S., Barreto S.L.T. (2011). Tabelas Brasileiras Para Aves e Suínos: Composição de Alimentos e Exigências Nutricionais.

[17] Zhu C., Guo C., Gao K., Wang L., Chen Z., Ma X.Y., Jiang Z.Y. (2017). Dietary arginine supplementation in multiparous sows during lactation improves the weight gain of suckling piglets. J. Integr. Agric..

[18] Nuntapaitoon M., Muns R., Theil P.K. (2018). Tummaruk, L-arginine supplementation in sow diet during late gestation decrease stillborn piglet, increase piglet birth weight and increase immunoglobulin G concentration in colostrum. Theriogenology.

[19] Pfaffl M.W., Tichopad A., Prgomet C., Neuvians T. (2004). Determination of stable housekeeping genes, differentially regulated target genes and sample integrity: BestKeeper—Excel-based tool using pair-wise correlations. Biotechnol. Lett..

[20] Livak K.J., Schmittgen T.D. (2001). Analysis of relative gene expression data using real time quantitative PCR and the 2^−^^ΔΔCt^ method. Methods.

[21] Lucy M.C., Safranski T.J. (2017). Heat stress in pregnant sows: Thermal responses and subsequent performance of sows and their offspring. Mol. Reprod. Dev..

[22] Ashworth C.J., Finch J.M., Page K.R., Nwagwu M.O., Mcardle H.J. (2001). Causes and consequences of fetal growth retardation in pigs. Reprod. (Camb. Engl.) Suppl..

[23] Reynolds L.P., Borowicz P.P., Vonnahme K.A., Johnson M.L., Grazul-Bilska A.T., Wallace J.M., Caton J.S., Redmer D.A. (2005). Animal models of placental angiogenesis. Placenta.

[24] Silva A., Dalto D., Lozano A., de Oliveira E., Gavioli D., de Oliveira J., Romero N., da Silva C. (2013). Differences in muscle characteristics of piglets related to the sow parity. Can. J. Anim. Sci..

[25] Nissen P., Jorgensen P.F., Oksbjerg N. (2004). Within-litter variation in muscle fiber characteristics, pig performance, and meat quality traits. J. Anim. Sci..

[26] Braun T., Gautel M. (2011). Transcriptional mechanisms regulating skeletal muscle differentiation, growth and homeostasis. Nat. Rev. Mol. Cell Biol..

[27] Semsarian C., Sutrave P., Richmond D.R., Graham R.M. (1999). Insulin-like growth factor (IGF-I) induces myotube hypertrophy associated with an increase in anaerobic glycolysis in a clonal skeletal-muscle cell model. Biochem. J..

[28] Barton-Davis E.R., Shoturma D.I., Sweeney H.L. (1999). Contribution of satellite cells to IGF-I induced hypertrophy of skeletal muscle. Acta Physiol. Scand..

[29] Grounds M.D. (2002). Reasons for the degeneration of ageing skeletal muscle: A central role for IGF-1 signaling. Biogerontology.

[30] Zanou N. (2013). Gailly, Skeletal muscle hypertrophy and regeneration: Interplay between the myogenic regulatory factors (MRFs) and insulin-like growth factors (IGFs) pathways. Cell. Mol. Life Sci..

[31] Glass D.J. (2005). Skeletal muscle hypertrophy and atrophy signaling pathways. Int. J. Biochem. Cell Biol..

[32] Aguiar A.F., Vechetti-Júnior I.J., Alves de Souza R.W., Castan E.P., Milanezi-Aguiar R.C., Padovani C.R., Carvalho R.F., Silva M.D.P. (2012). Myogenin, MyoD and IGF-I regulate muscle mass but not fiber-type conversion during resistance training in rats. Int. J. Sports Med..

[33] Wilson E.M., Hsieh M.M. (2003). Rotwein, Autocrine growth factor signaling by insulin-like growth factor-II mediates MyoD stimulated myocyte maturation. J. Biol. Chem..

[34] Aboalola D., Han V.K.M. (2017). Different effects of insulin-like growth factor-1 and insulin like growth factor-2 on myogenic differentiation of humam mesenchymal stem cells. Stem Cells Int..

[35] Oksbjerg N., Therkildsen M., Purslow P.P. (2017). Myogenesis and muscle growth and meat quality. New Aspects of Meat Quality.

[36] Bass B.E., Bradley C.L., Johnson Z.B., Zier-Rush C.E., Boyd R.D., Usry J.L., Maxwell C.V., Frank J.W. (2017). Influence of dietary L-arginine supplementation of sows during late pregnancy on piglet birth weight and sow and litter performance during lactation. J. Anim. Sci..

[37] Quesnel H., Quiniou N., Roy H., Lottin A., Boulot S., Gondret F. (2014). Supplying dextrose before insemination and L-arginine during the last third of pregnancy in sow diets: Effects on within-litter variation of piglet birth weight. J. Anim. Sci..

[38] Hong J., Fang L.H., Jeong J.H., Kim Y.Y. (2020). Effects of L-arginine supplementation during late gestation on reproductive performance, piglet uniformity, blood profiles, and milk composition in high prolific sows. Animals.

[39] Palencia J.Y.P., Lemes M.A.G., Garbossa C.A.P., Abreu M.L.T., Pereira L.J., Zangeronimo M.G. (2017). Arginine for gestating sows and foetal development: A systematic review. J. Physiol. Anim. Nutr..

[40] Wu X., Yin Y.L., Liu Y.Q., Liu X.D., Liu Z.Q., Li T.J., Huang R.L., Deng Z.Y. (2012). Effect of dietary arginine and *N*-carbamoylglutamate supplementation on reproduction and gene expression of eNOS, VEGFA and PlGF1 in placenta in late pregnancy of sows. Anim. Reprod. Sci..

[41] Liu X.D., Wu X., Yin Y.L., Liu Y.Q., Geng M.M., Yang H.S., Blachier F., Wu G.Y. (2012). Effects of dietary L-arginine or *N*-carbamylglutamate supplementation during late gestation of sows on the miR-15b/16, miR-221/222, VEGFA and eNOS expression in umbilical vein. Amino Acids.

[42] Che L., Yang P., Fang Z., Lin Y., Wu D. (2013). Effects of dietary arginine supplementation on reproductive performance and immunity of sows. Czech J. Anim. Sci..

[43] Wu G., Bazer F.W., Hu J., Johnson G.A., Spencer T.E. (2005). Polyamine synthesis from proline in the developing porcine placenta. Biol. Reprod..

[44] Kwon H., Wu G., Meininger C., Bazer F.W., Spencer T.E. (2004). Developmental changes in nitric oxide synthesis in the ovine placenta. Biol. Reprod..

[45] Wu G., Bazer F.W., Wallace J.M., Spencer T.E. (2006). Intrauterine growth retardation: Implications for the animal sciences. J. Anim. Sci..

[46] Reynolds L.P., Kirsch J.D., Kraft K.C., Redmer D.A. (1998). Time-course of the uterine response to estradiol-17b in ovariectomized ewes: Expression of angiogenic factors. Biol. Reprod..

[47] Vonnahme K.A., Wilson M.E., Ford S. (2001). Relationship between placental vascular endothelial growth factor expression and placental/endometrial vascularity in the pig. Biol. Reprod..

[48] Edgerton L.A., Erb R.E. (1971). Metabolites of progesterone and estrogen in domestic sow urine. I. Effect of pregnancy. J. Anim. Sci..

[49] Kensinger R.S., Collier R.J., Bazer F.W., Kraeling R.R. (1986). Effect of number of conceptuses on maternal hormone concentrations in the pig. J. Anim. Sci..

[50] Wu Z., Hou Y., Hu S., Bazer F.W., Meininger C.J., McNeal C.J., Wu G. (2016). Catabolism and safety of supplemental L-arginine in animals. Amino Acids.

[51] Holanda D.M., Marcolla C.S., Guimarães S.E.F., Neves M.M., Hausman G.J., Duarte M.S., Abreu M.L.T., Saraiva A. (2019). Dietary L-arginine supplementation increased mammary gland vascularity of lactating sows. Animal.

[52] Costa K.A., Saraiva A., Guimarães J.D., Marques D.B.D., Neves M.M., Barbosa L.M.R., Villadiego F.A.C., Veroneze R., de Oliveira L.F., Garcia I.S. (2019). Dietary L-arginine supplementation during early gestation of gilts affects conceptuses development. Theriogenology.

